# Management of Ciguatoxin Risk in Eastern Australia

**DOI:** 10.3390/toxins9110367

**Published:** 2017-11-14

**Authors:** Hazel Farrell, Shauna A. Murray, Anthony Zammit, Alan W. Edwards

**Affiliations:** 1 NSW Food Authority, 6 Avenue of the Americas, Newington, NSW 2127, Australia; anthony.zammit@foodauthority.nsw.gov.au (A.Z.); alan.edwards@foodauthority.nsw.gov.au (A.W.E.); 2 Climate Change Cluster (C3), University of Technology Sydney, 15 Broadway, Ultimo, NSW 2007, Australia; shauna.murray@uts.edu.au

**Keywords:** ciguatera fish poisoning, ciguatoxins, seafood borne illness, New South Wales, Australia, risk management, imported seafood, ciguatoxin analysis, clinical identification

## Abstract

Between 2014 and 2016, five cases of ciguatera fish poisoning (CFP), involving twenty four individuals, were linked to Spanish Mackerel (*Scomberomorus commerson*) caught in the coastal waters of the state of New South Wales (NSW) on the east coast of Australia. Previously, documented cases of CFP in NSW were few, and primarily linked to fish imported from other regions. Since 2015, thirteen individuals were affected across four additional CFP cases in NSW, linked to fish imported from tropical locations. The apparent increase in CFP in NSW from locally sourced catch, combined with the risk of CFP from imported fish, has highlighted several considerations that should be incorporated into risk management strategies to minimize CFP exposure for seafood consumers.

## 1. Introduction

Ciguatoxins (CTXs) are naturally occurring marine toxins, produced by species of the benthic dinoflagellate genus *Gambierdiscus*, common in the coastal reef environments of tropical and sub-tropical regions [1,2,3]. CTXs enter and accumulate in the food chain, as herbivorous fish graze on *Gambierdiscus* and, in turn, these fish are preyed upon by carnivorous fish. Seafood consumers risk contracting ciguatera fish poisoning (CFP) when they eat CTX-contaminated fish. In the human body, CTXs cause an array of gastrointestinal, neurological and cardiovascular symptoms, with a complex array of clinical manifestations including diarrhoea, vomiting, extremity paresthesias, temperature reversal, bradycardia, hypertension, coma and death in extreme cases [4,5,6,7,8]. Despite CFP being frequently misdiagnosed as other seafood related diseases (e.g., paralytic shellfish poisoning, neurotoxic shellfish poisoning, scombroid fish poisoning or chronic illnesses such as multiple sclerosis and chronic fatigue syndrome) [7,9,10], it is the most common non-bacterial seafood related illness worldwide [7,9].

Originally, CFP was considered an illness confined to tropical and subtropical latitudes, between 35° N and 35° S [11], however, information on the true distribution of the illness is limited [12]. In Australia, most reports of CFP have been derived from fish caught in the tropical state of Queensland [4,13]. The Northern Territory has a similar climate, although CFP cases have been lower, likely due to sparser human populations [14]. Further south, along the eastern coastline of Australia, in New South Wales (NSW, 28–38° S), there has been an apparent increase in CFP from both imported fish and Spanish Mackerel (*Scomberomorus commerson*) caught in the region’s coastal waters [15,16,17]. Since 2014, nine incidents of CFP have affected thirty seven individuals [15,16,17]. Prior to this, CFP outbreaks in NSW were rare from locally sourced fish. One outbreak in 2002 was linked to Spanish Mackerel caught in the north of the state [18] and imported fish were linked to two cases investigated by the State food regulator, the NSW Food Authority, in 2005 and 2009 (NSW Food Authority, unpublished data [16]). Earlier accounts of CFP outbreaks in the State are also few, and published accounts mainly refer to large outbreaks. In 1984, forty people were reported to have contracted CFP in Sydney [19]. In 1987, sixty three cases were involved in the largest documented single outbreak of CFP in Australia. The illnesses were linked to Spanish Mackerel sold in Sydney but caught near the Great Barrier Reef in Queensland [20]. A large CFP incident (>thirty cases) in Sydney in 1994 was also linked to a shipment of Spanish Mackerel from Queensland [21]. A further twenty six Sydney cases (three clusters) were linked to imported tropical reef fish during 1997 and 1998 [22]. 

An increase in reports of CFP in recent years could be due to several reasons. Changes in the distribution and abundance of both migratory pelagic fish species and *Gambierdiscus* may be occurring due to global climate change. Flowing southwards along the east coast of Australia, the East Australian Current is the dominant western boundary current, and has been strengthening in recent years, leading to the transport of tropical waters into more southern latitudes, impacting temperature (currently increasing at +0.75 °C per century [23]). *Gambierdiscus* is a largely understudied genus, and the impact of a changing global climate on CTX production and the distribution and intensity of *Gambierdiscus* species has yet to be fully established [24,25,26]. In addition, increasing accessibility for travel to tropical locations, along with higher demand, and subsequent imports of seafood products from new or expanding ciguatera ‘hot spots’ could result in notable increases in outbreaks of CFP in locations previously not known to be affected [9,24]. As NSW was traditionally an area with a relatively low occurrence of CFP, the recent incidents were assessed to determine common factors and knowledge gaps that should be incorporated into risk management strategies to minimize CFP exposure for seafood consumers.

## 2. Results

Five incidents of CFP, affecting twenty four individuals, were associated with Spanish Mackerel caught in NSW coastal waters between 2014 and 2016 (Table 1). Four reported incidents of CFP, affecting thirteen individuals, were associated with four different types of fish (Redthroat Emperor, Purple Rockcod, Green Jobfish and Grouper) imported into NSW between September 2015 and January 2017 (Table 1). Three of the fish were caught in and around international waters near the Capel Bank Seamount, off the coast of Queensland. The fourth fish (Grouper) was reportedly caught off the Queensland coast, between Cooktown and Lizard Island (Table 1).

Investigations into these incidents found five common factors:

### 2.1. In Some Cases It Was Difficult to Confirm the Species of Purchased Fish and Where They Were Caught

In all incidents where the fish was caught in NSW, the fish was identified by the fisher as Spanish Mackerel, and the catch date coincided with the peak fishing season for this species (February–April). Details of the imported fish were more difficult to ascertain (Table 2). In two of the investigations, the purchaser could only provide a general indication of where the fish was purchased. Receipts were not available in three of the four investigations. Only one of the purchasers could recall a name/descriptor of the fish they purchased as Kingfish (Incident 6) but that information was later found to be incorrect during the product trace back investigation when the product was identified as Green Jobfish (Table 1). For one investigation, the catch details could not be linked to catch records. In addition, when available, catch records only reported a general location. Thus, the process of identifying the species of fish implicated and where it was caught was inherently time consuming and imprecise. In most cases the species of the implicated fish was determined through comparing the purchaser’s recollection of their purchase with documents relating to the sale of fish along the supply chain from the retailer back to the fisher. This involved: re-interviewing consumers to confirm purchase details; reviewing photographs of the implicated fish (where available) to confirm the implicated species; a trace-back through up to four suppliers; and a review of catch records (where available, Table 2). The time taken to identify the fish varied, the quickest being within 48 h.

### 2.2. Samples of the Cooked Meal or Fish Were Not Always Available

Fish samples were available for testing from four of the five CFP cases linked to Spanish Mackerel. Pacific ciguatoxin-1B (P-CTX-1B) was detected between 0.1 and 1 μg/kg (Table 1 [16,17]). For the imported fish investigations, fish/meal samples were only available for testing from two of the four incidents (Table 2). P-CTX-1B was detected in samples relating to one of those incidents. P-CTX-1B was detected in samples of fish from the same batch as the fish implicated in two of the investigations.

### 2.3. It Was Difficult to Determine the Level of Ciguatoxin in the Fish

At the time of analyses, P-CTX-1B was the only available reference material, and it is expected that other ciguatoxin analogs were also present in these fish samples. A strong indication of this was the ongoing symptoms of two of the cases related to one incident (Incident 9, Table 2 and Table 3), despite ciguatoxin not being detected in the samples provided. Four months after the initial illness, one of these cases described their symptoms as: “peripheral neurologic symptoms such as paresthesia in the extremities, paradoxical temperature reversal (an alteration or “reversal” of hot/cold temperature perception) and pruritus and neuropsychiatric symptoms such as malaise, depression, generalized fatigue as well as neurologic disorders such as anxiety which caused the development of nerve pain and other problems e.g., bradycardia (slow heart rate) and sweating”.

### 2.4. There Appeared to Be an Association between the Severity of Illness and How a Fish Meal Was Prepared and Consumed

Cases who consumed the viscera and/or head of the implicated fish that had been cooked in a soup or stew reported more severe symptoms. Those who consumed multiple or large portions of the same fish meal also experienced more severe symptoms. This pattern was observed across and within incident clusters (Table 3). Prolonged symptoms were observed for up to at least seven months following consumption of the implicated fish meal.

### 2.5. Affected Consumers Expressed Frustration at What They Described as “Poor” and “Inadequate” Information about Ciguatera Fish Poisoning, Its Effects and Management

While general advice to consumers is available in the form of factsheets (e.g., [27,28]), during interviews, many of the cases involved were not previously aware of CFP. Affected individuals expressed frustration at lack of support and advice on how to minimize potential further ciguatoxin exposure.

## 3. Discussion

The risk caused by ciguatoxins is not new. Traditionally, in tropical regions where incidence of CFP is high, there appears to be a general acceptance of the illness and its symptoms [29]. Yet the effects of CFP can be enduring and debilitating. They are also poorly understood. In addition, although CFP cases are generally underreported they have recently been identified in regions where CFP was previously undocumented, rare or linked to imported fish only (e.g., Australia [17], Canary Islands [30,31], Gulf of Mexico [32] and Japan [33]). There has also been a spike in reported cases linked to imported fish in regions in which CFP is not endemic (e.g., Germany [34,35], NSW, Australia [15], France [36], New Zealand [37]). Historically, reports of CFP linked to imported fish in NSW were associated with high case numbers [19,20,21,22]. As larger illness outbreaks are likely to garner more attention, it was difficult to ascertain whether CFP incidents with fewer cases did occur previously and were either not reported or misdiagnosed. Regardless, the new incidents of CFP in NSW highlighted knowledge gaps that limited assessment and management of ciguatoxin risk.

### 3.1. Identification of Implicated Fish Species and Catch Location Was Problematic

More than 90 species of fish have been linked to CFP, most of these species originating from tropical or sub-tropical locations [26]. The NSW cases demonstrated the importance of traceability through the supply chain in addressing biotoxin risks. Any industry response to this risk is limited by the ability to identify where and when the implicated fish was caught. During the investigations in NSW, it was easier to conduct trace back analysis and confirm species identity when the implicated fish (Spanish Mackerel) was caught and consumed by the fisher in NSW. In most of these incidents, the fish were caught by recreational fishers who shared their catch with family or friends. Trace back analysis of incidents linked to imported fish were complicated by four factors: none of the purchasers could accurately identify the type of fish they consumed; most purchasers did not retain any proof of purchase; recollection of purchase location was unclear; and, the process of confirming catch location through suppliers and fishers was time consuming. In practice, the time taken to identify fish that may pose a risk of CFP limits risk management. This is because the implicated fish or catch would usually have been consumed by the time a trace-back had been completed. In addition, it was not possible to confirm whether other fish from the same batch posed a risk. Improvements in labelling and traceability of seafood, would reduce the time taken to distinguish and track fish implicated in illnesses. Improved traceability would also lead to quicker and more accurate mapping of ciguatera “hot spot” locations, and hence improved identification and management of catch areas. It may also lead to efficient use of food withdrawal and recall procedures to better manage CFP risk.

### 3.2. The Prevalence of Ciguatoxins in Seafood Is Not Known

There are insufficient data to determine the prevalence of ciguatoxins and the concentrations at which they pose a problem for human health [7,9,13,38,39]. This knowledge gap could be significantly addressed through improved collection of samples from reported cases. For this to occur, it is vital that two strategies are put in place. The first to ensure potential CFP cases are identified. The second to ensure that, where available, meal samples from those cases are collected. Improved communication pathways between health professionals and regulators for suspected medical cases can facilitate this.

For this to happen community awareness of the increasing likelihood of CFP incidents needs to improve. This would include increased awareness that: CFP is not seasonal (illnesses investigated in NSW were reported at different times throughout the year); all age groups can be affected at different degrees of severity depending on consumption factors and each individual consumer’s overall health; and previous exposure to ciguatoxins of an individual can contribute to more severe effects. In addition, Health professionals’ awareness of CFP is key to early diagnosis. Two primary indicators found in the NSW cases were temperature dysesthesia and whether those symptoms were associated with consumption of the head or offal of the fish, particularly if it was cooked in a soup or stew [15].

While CFP can be misdiagnosed, potential cases can be identified and sample collection encouraged. One case reported taking samples to a hospital emergency department but these samples were not retained for testing. In addition, sampling could also be improved through innovative approaches to that problem. A recent citizen science driven project detected P-CTX-1B in flesh (*n* = 1) and liver (*n* = 5) samples from 71 Spanish Mackerel caught in NSW coastal waters [38]. This was the first baseline study of ciguatoxins in NSW waters.

### 3.3. How Do We Accurately Determine the Level of Ciguatoxins in Fish?

There are more than 23 ciguatoxin analogues that have been identified to date, and several others that have been detected but are as yet uncharacterized [40,41]. There is very little knowledge of their cumulative impact, and testing of their comparative toxicities has been minimal [41]. The availability of standardized reference material is currently limited to only a few researchers worldwide (i.e., [41]). Therefore, any routine testing for ciguatoxins is unlikely to provide reliable data on the nature and extent of the risk posed by the toxin. This is one of the main reasons why a regulatory limit for ciguatoxins has not been established. The current US FDA guidance levels are 0.01 μg/kg ciguatoxin equivalent for Pacific CTX [11]. Concentrations of P-CTX-1B in NSW illness cases have been up to 2 orders of magnitude higher [15,17]. In previous research, illnesses have been reported due to the consumption of fish flesh containing between 0.08 and 0.1 μg/kg of Pacific CTX-1 [13].

In the NSW investigations, the samples analyzed were often from a different part of, or the same batch of, the fish consumed. Variability of toxin content between different parts of the fish is expected, particularly with respect to higher concentration of CTXs in fish viscera [38,42]. In studies of Spanish Mackerel, it has been found that liver samples on average contained approximately five times higher toxin levels than muscle (flesh) samples [38]. However, multiple CTXs may co-occur in fish samples, including in Spanish Mackerel in Australia, therefore, it is not possible to determine overall toxicity of a fish in the absence of toxin standards for all CTX analogs [43].

In order to ensure total toxicity can be taken into account, it is important that additional toxin reference materials, such as the sixteen toxin analog standards that were utilized in a study of ciguatoxins in fish in Japan [41], are made available to the broader community. Important additions to LCMS based assays, such as receptor binding assays or the neuroblastoma assay for CTXs (i.e., [44,45]), cannot be commercially developed without such reference material. The UNESCO Intergovernmental Oceanographic Commission’s Global Ciguatera Strategy 2015–2019 was established in order to strengthen research into CFP around the world. This strategy focuses on all aspects of the causes of CFP, including a greater understanding of the biological processes behind CFP, such as the identification and enumeration of *Gambierdiscus* species, in order to identify potential CFP hotspots. For example, a highly significant positive correlation has been found between times of peak density of *Gambierdiscus polynesiensis* and CFP incidences due to reef fish from a geographical area, with a lag time of approximately three months, in a study from French Polynesia [46]. Studies from the Caribbean have similarly shown that the abundance of *Gambierdiscus* species correlate significantly with CFP incidences in a region [47].

### 3.4. How Can We Alleviate the Concerns of Consumers and Industry Regarding CFP?

The difficulties in managing the risk of CFP are well documented (refer to reviews by Friedman et al. [7,9] and Rodgers and Diogène [48]). Promoting awareness of CFP is essential, to assist consumers to minimize their exposure to the illness. It is also a key aspect in minimizing risk to industry. Industry awareness of CFP appears to be high. In incident 5, the consumer reported the incident to the supplier of the fish who promptly passed that information to the regulator. Despite this, the concern with the apparent increase of CFP in previously low-risk areas is not limited to the impact CFP has on affected consumers and their families. An increase in CFP cases may also lead to legal action and a general loss of confidence in the seafood industry. An example is the litigation that followed a CFP incident linked to a 16.2 kg Maori Wrasse fish, a fish “not generally accepted for sale in Queensland”, that was imported into Victoria from Trunk Reef in Queensland in 1997 [49]. A consumer who suffered CFP after consuming part of that fish made a legal claim against the restaurant for damages. The restaurant in turn included the supplier of the fish in those legal proceedings. The trial judge found that both the restaurant and the supplier of the fish were negligent and that the fish served was not of merchantable quality. In 2003, some six years after the incident, legal issues had not been resolved. Representatives for the supplier of the fish and the restaurant that served it were in the Victorian Supreme Court arguing liability. The court noted: “The fish was a Maori Wrasse. There was undisputed evidence at the trial that that is” “a species of fish known to cause ciguatera poisoning”, that “[t]here is no part of the fish that is safe for its consumption” and that “there is no reliable testing available that can be used to detect ciguatera toxins prior to consumption” [50]. The findings of the court contrast with the conclusion of the government health department that investigated the incident that: “The low prevalence of the toxin in fish (in areas known to have contaminated fish, 1 in 5000 coral trout is estimated to be toxic [4]) and the lack of a simple routine test for the toxin means consumer education is important. We identified a need to inform Asian restaurateurs of the risk of ciguatera fish poisoning and the need to avoid large reef fish, especially the head and other viscera” [49]. This is not surprising as the Court was considering an individual case of civil liability, not the broader public health policy considerations that were faced by the regulator. It highlights the complexity in managing this risk.

In summary, not only does CFP impact consumers but a failure to implement practical measures to reduce the risk of CFP can result in an added burden of cost for industry and the overall economy [9,51]. It can result in loss of confidence in products or industry sectors. It can lead to increased insurance premiums and operating costs. It is acknowledged that co-operative arrangements where industry and regulators work together to minimize risk can be cost effective in addressing risk [52]. So, while establishment of a monitoring program for CTXs is not currently feasible, existing strategies for CFP risk management: traceability; recall and education could be reviewed by both regulators and industry to identify improvements that could further reduce risk to consumers. For example, improvements in traceability, identification and control of high risk fish and CFP ‘hot spots’ could lead to agreed systems that form the basis of a ‘due diligence’ defense to statutory offences. (Note: Under Section 22 of Annexure A of the Model Food Act, which has been adopted by each Australian State and Territory: “In any proceedings … it is a defence if it is proved that the person took all reasonable precautions and exercised all due diligence to prevent the commission of the offence by the person or by another person under the person’s control.” [53]) They may also go some way to demonstrating that a supplier has discharged their duty of care to a consumer.

General advice to consumers remains the main strategy addressing CFP risk. In NSW, publication of educational advice to recreational fishers has coincided with the annual Spanish Mackerel fishing season (February–April) in the State’s North [54]. While a recent study by Kohli et al. [38] found that there was no link between the size of Spanish Mackerel and the amount of P-CTX-1B in the fish tissues, reported illness cases have been linked to larger Spanish Mackerel (>10 kg) [16,17]. While CFP illnesses were not reported during the 2017 Spanish Mackerel fishing season in NSW, continued outreach to fishing groups should be maintained. While the risk posed by imported fish is not seasonal, not all fish from a ciguatera “hotspot” will carry ciguatoxins. Whilst it is noted that not all incidents recently investigated in NSW specified whether the fish head was included in the meal, there is a strong indication that this cooking method may increase the risk of CFP. Due to individual case variability within the four incidents reported here, it is difficult to confirm whether the cooking method of including the fish head and/or viscera in a stew, casserole or soup increased the likelihood of exposure. It does however seem likely, as ciguatoxins are more concentrated in the viscera and/or head of contaminated fish [38,42] and there are several reported cases which highlight severe symptoms from consuming soup, stew or broth made with ciguatoxin-contaminated fish [6,30,55]. Advice to consumers should note the increased risk posed by consuming:

- any portion of a fish that could contain CTX that has been cooked in a soup, stew or broth with the head and/or viscera of that fish; or
- multiple serves of the same fish meal.

## 4. Conclusions

The nine cases investigated highlighted that both imported fish from tropical locations and Spanish Mackerel caught in NSW are potential causes of CFP for NSW consumers. The investigative approach was the same for either source of fish. The strategies in place to manage ciguatoxin risk are dependent upon accurate information about the nature and extent of the risk. During the investigations, it was apparent that confirmation of diagnosis, fish species and origin was difficult, and often meal samples were not obtainable. Key information to be ascertained from a CFP investigation relates to:

1. Where the fish was caught;
2. The species of the fish;
3. What symptoms are reported;
4. The concentration and distribution of the toxin in the fish;
5. How the fish was processed and cooked;
6. How the fish was eaten.

CFP is a concern for seafood consumers, industry and regulators. As regulators, the most desirable outcome is to establish a cost effective, reliable, routine test for ciguatoxins to protect both consumers and the seafood industry. In lieu of this, risk management is reliant upon regulators and industry working to improve existing strategies including communication campaigns for consumers, improved catch traceability, improved regulation of high risk catch sites and the support of targeted research to improve testing capabilities.

## 5. Materials and Methods

In NSW, surveillance of foodborne illness is monitored by NSW Health and the NSW Food Authority. Nine CFP incidents occurred in NSW between 2014 and 2017. Where possible, each investigation involved a trace-back to supplier and catch locations, interviewing those affected to determine their symptoms, the amount of fish consumed, how the fish was prepared and consumed and whether there was a history of the individual being previously exposed to CFP [15,16,17]. These investigations were reviewed and compared to determine gaps in the investigative process and to identify barriers and limitations to CFP risk management approaches. When possible, samples of the implicated fish or meal leftovers were collected and tested for P-CTX-1B. This was the only CTX reference material available at the time of testing and it is expected that several other CTX compounds were present in the samples. Analysis for P-CTX-1B was via liquid chromatography mass spectrometry [38]. Samples from incidents 1–3 were analyzed at the Cawthron Institute, New Zealand. As capacity for testing was established at the Australian Research Facility for Marine Microbial Biotoxins, Sydney in 2015, samples from incidents 4–9 were analyzed at that facility.

## Figures and Tables

**Table 1:** Summary of reported CFP incidents in NSW since 2015 [15,16,17].

Incident	Date	Cases	Fish Species/Origin	P-CTX-1B (μg kg^−1^)
1	Feb. 2014	4	Spanish Mackerel/Evans Head, NSW	nd, 0.6, 1
2	Mar. 2014	9	Spanish Mackerel/Scotts Head, NSW	0.4
3	Apr. 2015	4	Spanish Mackerel/South West Rocks, NSW	n/a
4	Sept. 2015	3	Redthroat Emperor/Capel Bank Seamount	0.023
5	Sept. 2015	1	Purple Rockcod/Capel Bank Seamount	0.069 *
6	Feb. 2016	5	Green Jobfish/Capel Bank Seamount	0.006 *, 0.036 *, 0.02 *
7	Mar. 2016	3	Spanish Mackerel/Crowdy Head, NSW	0.93
8	Apr. 2016	4	Spanish Mackerel/Crescent Head, NSW	0.11, 0.37
9	Jan. 2017	4	Grouper/Between Cooktown and Lizard Is.	nd

* Batch samples were tested when meal samples were not available. n/a = not available. nd = not detectable.

**Table 2:** Summary of available information from four CFP incidents linked to imported fish in NSW since 2015 [15].

Incident	Purchase Receipt Available	Meal Sample Available	Positive for Pacific Ciguatoxin-1B	Fish Type Known by Consumer	Catch Details Linked to Records
4	No	Yes	Yes	No	Yes
5	No	No-batch sample	Yes	No	Yes
6	No	No-batch sample	Yes	No	Yes
9	Yes	Yes	No	No	No

**Table 3:** Summary of meal types and symptoms associated with CFP incidents in NSW since 2015 [15,16,17].

Incident	Meal Type/Preparation	Cases *	Symptom Onset Time	Comments	Fish Type/Origin
1	Fish trimmings used for fish cakes.	4/7	3–4 h.	One individual, with most severe initial symptoms, had neurological symptoms up to seven months later.	Spanish Mackerel/NSW
2	Fillet steaks cooked on barbeque.	9/9	1–4 h.	One individual reported a persistent metallic taste up to two months later.	Spanish Mackerel/NSW
3	Unknown.	4	Unknown.	At least one individual was hospitalised.	Spanish Mackerel/NSW
4	Fish was ‘butterflied’ (spine removed and eviscerated). Baked in a casserole with head on.	3/3	‘Shortly’ after eating the head of the fish.	One individual ate head of the fish and reported to hospital three days later with worsening symptoms. Other two individuals ate only a small amount and described feeling generally unwell.	Redthroat Emperor/Imported
5	Unknown.	1/1	Unknown.	No additional comments available.	Purple Rock-cod/Imported
6	Fish soup.	5/6	5.5 h.	Individuals who ate the head of the fish had more severe symptoms than those who consumed fish fillet only.	Green Jobfish/Imported
7	Fish fillets.	3	3 h.	Symptoms included reverse temperature sensation, tingling/numbness in hands and around mouth, chest tightness/pain, diarrhoea and nausea.	Spanish Mackerel/NSW
8	Fish fillets.	4	Not confirmed.	Symptoms included numbness/tingling around mouth/hands, loose stool, aching teeth, reverse temp sensation.	Spanish Mackerel/NSW
9	Whole fish was prepared three ways: steamed, fillet and in stew (hotpot).	4/11	Not confirmed.	Two individuals, with more severe symptoms, which persisted for at least four weeks, consumed two portions of stew (hotpot) over two days. The other two individuals, whose symptoms were less severe, consumed only one serve of stew (hotpot) each. Other individuals present did not eat the stew (hotpot).	Grouper/Imported

* Cases refers to the number of individuals that exhibited CFP symptoms. Where available, the total number of individuals who consumed the fish meal is provided.
